# How to Conduct a Dental Practice

**Published:** 1869-08

**Authors:** W. F. Morrill


### HOW TO CONDUCT A DENTAL PRACTICE.

BY W. F. MORRILL, D. D. S.

Read before the Indiana State Society.

"Men are sometimes masters of their fate."—*Shakspeare.*

It is generally conceded that every one who has practiced Dentistry for any considerable number of years, understands best how to manage his own affairs. To be able to give advice, or speak with confidence on a subject, engrossing so large a share of your own experiences, pre-supposes an ample stock of my own; otherwise, what is said must be considered only as vague theories, and not of much value. In support, then, of what I may offer, allow me to say that my Dental practice extends over a score of years, back to 1849. In the swift currents of our prosperous profession, this venerableness of years ought to entitle me to speak with some degree of boldness and plainness; and with such experiences as I may draw from, having no very gay botanical plants scattered along my pathway, you are welcome to weigh, compare and possibly to digest them.

When Oliver Cromwell returned from his campaign, he entered the studio of an artist to have his portrait painted, and, throwing down his hat, he exclaimed, "paint the wrinkles!" Possibly, in this attempt of mine to serve you with a faithful copy, I may be compelled, in order that my delineations of Dental practice shall be better understood, to add a few wrinkles. And, if in these outlines, there be a close resemblance—a striking likeness—to those wrinkles, which any of you here present may bear, remember you can yet amend your professional campaign, and avoid the blush they may occasion you.

Without further prelude, let me say, as a means toward conducting a Dental practice right, much is accomplished by organizing into societies, and meeting together regularly and periodically. This frequent interchange of sentiment and ideas result in good. These are opportunities for gathering useful hints, and strengthening our professional manhood. By mingling here pleasantly, we promote a more general impulse and systematic direction in research after scientific knowledge; our prejudices are softened down; our antipathies are obliterated, and an influence at once salutary and ennobling is exerted. We can not keep ourselves aloof from these periodical gatherings without losing the zest and incentives to progress, and without deteriorating in our acquaintance with various methods of practice. Those who profess to see no advantages to be derived from this source, and are inimical to these organizations, may often be found specimens of bigotry, self-conceit, and narrow-mindedness. I recollect an instance of the kind. An old practitioner who never had been present at one of these meetings, received an invitation, and to the surprise of those who knew him, he came. His Websterian proportions impressed me favorably. I anticipated a treat from his gathered years of experience and ripened wisdom; but it was reserved for me to be disappointed. He no sooner rose to speak than he began to display a bundle of conceits—a strange medley of practice—rarely, if ever before witnessed. He showered on us some of the most stupendous physiological problems and monstrosities the keenest and most astute ever saw. He unfolded the pathological conditions of his remarkable cases with surprising gusto. But his dignity was mere pomposity; his methods, so marvelous to himself and us, possessed more of necromancy than science; his performances were so incredible and self-laudatory, they seem to belong more to an age of superstition than to a modern day. On taking his seat, the sharp arrows of inquiry that immediately followed into his *adroit* methods of practice, so disconcerted his equanimity, that he lost his temper and his manners, and stalked defiantly out of the hall. This absenting himself was a great loss to him, not to the society. Had he remained, his mortification and chagrin might have been turned into an awakened sense of his own ignorance and stupendous folly.

It is hardly possible, not probable, any one man can master all that is worth knowing in Dental practice, and then shut himself up like a snail or a hybernating *ursus*. The wisest thing a turtle does is to stick his head out of his shell; it is good for the turtle, and complimentary to other turtles. We are presumed to gain by the tried experiences of others—especially so, when the qualifications are superior to our own. It is here we receive the records of those in the field of practice; we investigate their facts, observations, and experiences, to see how far they tally with our own. These are some of the advantages flowing out of organizations beneficial to the conduct of a Dental practice. Every practitioner who has been regular in his attendance here, who extends his courtesies, and enjoys the sweet amenities of these occasions, can not fail afterwards to improve his Dental practice.

Among the essentials that seem to form a connecting link in the plan of my subject, but on which I am not very competent to speak, is correct taste in Dental practice. That this faculty is of great practical importance, I need not suggest. On it hinge the beauty and excellence of all Dental operations. Without it, discouragements, failures and disasters are the natural consequences. Out of the vast number of Dental operations made, only a few display the exquisite virtues of this great element. If proof were wanted of its dearth, we have but to turn our eyes toward the countless number of unsightly specimens, everywhere exhibited, by contour of features, in mouths with a dumpling-like puff, or a lemon-like squeeze, showing the hand that made those teeth was not divine, but very false indeed!

Now, what do we understand by taste? " It is, says Webster, "judgment, discernment, a power of perceiving and relishing excellence in human performances; that faculty of discerning beauty, order, congruity, proportion; or whatever else constitutes excellence, particularly in art and science." We are valued in society, we are estimated by our co-laborers, just in proportion as we possess this esthetical faculty; and it is, therefore, of the highest importance to have it. It is, moreover, true, we have comparatively few models in Dental art and science by which to form a correct taste. In operative Dentistry, especially, the fillings regarded as first class, displaying a versatility of art, and unexceptionable good taste, are not so numerous as they should be. Many practitioners yet move along in certain ruts or grooves of traditionary methods, whereby they leave their paternal impress on nearly every operation they make. To patriarchs and prophets from whom they received their revelations, they cling with a persistence and fidelity marvelous to behold.

For the display of this attribute, deservedly called taste, the field of Dentistry is wide and plenteous. Its value so inestimable in the management of a Dental practice that I proceed to show how we may improve its properties. This may be done by familiarizing ourselves with those models and methods conceded to be superior and regarded as perfect as human performances can make them. Until this acquaintance is established no one should suffer egotism to lull him into a fancied security of having to any very great extent this faculty. Its possession involves culture, expenditure of time, energy, diligence, and a keen relish for the beautiful in art and science. If you are exemplars of taste, the character of your operations will show it; and on the character of your operations will be your pre-requisite duty or else the management of your Dental practice at the outset will be poorly begun.

Closely allied and inseparably connected with the cultivation of taste, is art. Its principles in Dental practice are not generally well enough understood to make either great boastings or pretensions. Nevertheless, every operation in Dentistry has appropriate art. "Expression," says Powers, "is art." "Form is the essence of expression, and conveys precisely the signification of the thing." How much of this essence of expression, do we see in a large majority of artificial dentures, or in fillings, which have not restored the complete loss of the tooth substance? In the oral cavities there are many hideous deformities. Nature there sometimes plays her ugliest pranks. Unless we can remedy and restore, we falsify and degrade our vocation. Into these new creations of our own we are called on to mould regularity, harmony, proportion, comliness and beauty; "to put into them life and seeming intellect, where before were decay and unbreathing forms." We are compelled to improve on nature, or else, into the sphere of art we do not rise. And "out of the supernatural is art born." If we would command success in the domain of art, we must endeavor to acquaint ourselves with the principles which underlie its foundation; and, with a trained eye and practiced hand, we shall see rising into triumphs and perfections our ideal embodiments, that add honor and lustre to our profession.

Some larger and lesser rules for the conduct of a Dental practice enter into my plans, which I now proceed to notice. Certain virtues are of great necessity to a Dentist, and certain vices are to be guarded against. Our duty involves the practice of every virtue, and the shunning of every vice. Our relations to our patients are intimate, delicate, confiding in their character, and must need be preserved inviolable and intact. We can not be too punctillious in what occurs in the presence of patients. Conversations, manners, habits, are all carried home, there to form the subjects for comment and scrutiny. See to it that these be without blemish. Your manners should not have an air of harshness, nor pevishness, nor rudeness. Be neither frivolous nor effeminate. If you wish to spur up your patient to more resolution and courage, do it without provoking contempt. Gain his confidence by telling him the truth, if any representations are to be made. Interest your patients when circumstances will admit, on topics genial and pleasant. If they desire information on the teeth, give it to them in such doses as they will be able to bear. Season your teachings with a trace of spice, and avoid the use of technical terms. Avoid astonishing your patients with two things—your remarks and your performances. Truth, and no lying, should guild conversation and the character of your operations. Be guarded in your promises if it fails, the mischief can not be easily repaired by apologies.

The rooms of your office should be free as possible from indicating the nature of your calling. They should be plain, neat and comfortable, yet strictly conform to a discreet taste. Any display above this, excessive ornament, colored anatomical lithographs, skulls, specimens of old teeth in bottles, or relics of any kind, savoring of your occupation, not excepting even specimens of artificial teeth in show cases, should be concealed from public eye. If you have any of these, let them be brought forth only on rare occasions. It is not necessary for these many signboards to your vocation; nor will they furnish additional proof that you are either profound or proficient. Napkins soiled, spots of blood any where visible, instruments clinging with debris, or any taint saluting your patients may prevent a repetition of their visits to your office. While operating, have as few mishaps occur as possible; make your guards secure that no instruments shall slip into the soft parts—an injury is scarcely to be atoned for by apologies.

Attentions to your own personal neatness and cleanliness are not to be slighted or neglected. Don Quixote's advice to Sancho Panza when about becoming a governor of an island, respecting the cleanliness of his finger nails, and other admonitions quaint and homely, would not be impertinent to our day. A due regard for the graces of neatness and cleanliness adds much towards refinement and godliness. Finery, gentility, suited to a bar-tender, dry goods clerk, or gambler, would hardly be appropriate for a gentleman, and a Dentist. For the fashion of dress, let it be dictated by discreet taste—tidy, and a happy medium between extremes. Professional emblems are pretty, but queer taste. The fumes of liquor or smoke in your office will put your practice on the wane. There is an impressible conflict—a moral antagonism—between Dental practice and foul habits. Let purity be your personal aroma. The office is sometimes a convenient place for friends to drop in and chat. Have no long tarryings there. Ladies do not like to enter where loungers congregate. Freeze out these interlopers by words or looks. Have your time so arranged that each hour may be profitably employed. Stick close to your office the first ten years; after that the habit becomes fixed, and you need have no fears afterwards.

I desire to call your notice to another habit, almost too trivial to mention, yet, as you will perceive, it deserves a passing notice. I allude to a disposition to complain and repine over the mishaps and perplexities of life. A tolerably fair acquaintance with Dentists, induces me to believe the invalid corps among them is large. If we may credit the reports of themselves, it is surprising so many of them are above ground. The maladies of which they complain, strange to say, are not of such a malignant type as to carry any of them off; nor have our "guides to health" proved sufficiently efficacious in restoring them. Indeed, it would hardly be safe to say that their bodily ailments are chiefly under the heads of rheumatism, dyspepsia, headaches, backaches and kindred complaints; but most probably they are the result of vexations, frictions of a burdensome Dental practice, the increasing cares of their domestic circle, that make these repinings their staple topic. But however ready told they are, and the sympathetic tear be ready to fall "in piteous chase," no alleviation of distresses ever come by these complainings. Make labor—well directed industry—bring you heroic doses of greenbacks, and my word for it, the ills of life will tone down, and you will not be a *weeping-willow* of the profession. A fair credit in bank is the grandest prophylactic against sourness, ill temper, and crabbiness the world ever saw. It makes a lengthened, doleful, persimon face round out fat, jolly, mirthful and rollicking. It is the completest antacid materia medica can offer.

But, confess it we must, Dentists, like preachers and poets mostly die poor, and die young. They do not burn to "the socket," though money burns in their pocket. Free, generous, hospitable, refined in their sensibilities and cultivation, they are not apt to become rich. Strange as it may appear between the art of money keeping, and the art of Dentistry, there seems an incompatibility.

Finances is an earnest, central fact ever staring us in the face, whether in our waking moments, or in the whispering slumbers of the night. It steals in on us like some insolent and unwelcome ghost; nor can we escape or flee from this all absorbing phantom, however imperious he may be in his demands.

The expenses of home, office, state, municipal, county, internal revenue, taxes, patent royalties, and a thousand others, ever throng our minds for solution. Can you expect to meet these obligations promptly, and have them liquidated by charging low fees? If you can, your abilities as financiers surpass those whom we have of late seen vacate the departments of the United States Treasury. Lowness of fees, as a rule of practice, is not honest to yourself, nor respectable to others. A practitioner who adopts low fees, degrades his calling below the barber who leeches, cups and draws teeth. Under sharp competition, I am aware how hard and difficult it is to conduct a practice within the strict rules of propriety and the "code;" nevertheless, it rarely pays to lose our self-respect, professional pride, and dignity, by doing a mean and contemptible act.

There is also another practice to which some are addicted that is alike disreputable and ungenerous. I allude to advertising. I regret to say there is a class of Dentists who have a propensity to appear in print, thereby hoping to make themselves suddenly rich; in both of these enterprises they fail. They get up some trap, or what they consider a new fangled notion, and forthwith they have affixed the seal of a patent upon it; then, in huge "caps," cuts and circulars they herald it to the public. These are, *false teeth on new base.* They are certain to supersede rubber or any other elastic material. These presumptuous advertisers are fond of telling the public how gifted they are; how superior and profoundly unique are their methods compared with those of their neighbors. Their discoveries are all their own; and everybody else is in blissful ignorance of them—"springs to catch wood-cock." The people, attracted by such humbugs, are usually an indifferent and unpleasant class; they will not stick fast friends to any one. To such ambitions as these, I say, squelch them. Remember as you have received good gifts yourselves, so likewise you should give good gifts in return. And what proportion can you even then repay to cancel the great debt of gratitude you owe? *Respect your profession* by shouldering its burdens, by making contributions to science and art, by living upright and honorable to the highest code of ethics, and by doing no act which will lower you in your own estimate, or in that of others.

Dental practice, to some, is a fickle God. In spite of all efforts it sometimes takes wing. Owing to circumstances, of instability of finances and commercial affairs, of improper means employed by mountebanks, or other attendant peculiarities of your own, which I can not presume to conjecture, these interludes, happen. It does not appear merciful nor kind, but you accept the situation. Moreover, there occurs a long blank to some, separating them from the object of their ambition. Many are lost by the wayside. They have

"Waited with a martyr smile
Hope ever whispering, *yet a little while;*
Too proud to stoop beneath thy noble aim,
While prostrate meanness crawls to wealth and fame;
Thou all unfriended as thy blossoms fade,
In the chill circle of thy seniors shade;
Go, spurn the art that every boon denies,
Till age sits glossy in thy sunken eyes;
Go, scorn the treasury that withholds its store,
'Till hope grows cold, and blessings bless no more."

If these inter-regnums of leisure come to you, devote yourself to the calm pursuit of Dental truths, to a liberal range of knowledge which busy periods do not afford, and these trying days will roll away, and the fulfillment of the golden harvest so long in expectancy may yet be yours.

Remember, in the management of Dental practice, *pluck* is a great element of strength. It denotes concentrated energy, will, courage, prompt decision. It bears the relation to the ordinary propelling powers of man, that an additional steamboiler does to an engine. It may be innate, born the same as poesy, sculpture, or painting, and it may be cultivated and nursed into vigor and strength. At all events, whatever may be your accomplishments as a dextrous operator, whatever may be your researches after science, whatever may be the estimate you are held in society, whatever may be your qualities of head and heart, whatever may be the ordeals or experiences through which you may pass, if you have not this mental force you are a comparative failure. Select from those brilliant patterns of thrift who leave their marks on the world, and you have models from which you can study and work out that which gave those men success, and endeavor to incorporate into your daily practice such parts as may be practicable to your own condition. But be not allured from your professional pathway by schemes of speculation, nor by the tempting and debasing emoluments of office. These are thrice apt to bring ruin and disaster. "Small and steady gains ensure tranquility of mind," is a pocket piece worth carrying.

Gentlemen, let us bear the ills of life with the same constancy with which others endure them, accept our manly part, hold our own and ask no more.

